# Short-term sleep deprivation leads to decreased systemic redox metabolites and altered epigenetic status

**DOI:** 10.1371/journal.pone.0181978

**Published:** 2017-07-24

**Authors:** Malav S. Trivedi, Dana Holger, Anh Tuyet Bui, Travis J. A. Craddock, Jaime L. Tartar

**Affiliations:** 1 Department of Pharmaceutical Sciences, College of Pharmacy, Nova Southeastern University, Fort Lauderdale, Florida, United States of America; 2 Department of Psychology & Neuroscience, Computer Science, and Clinical Immunology, Nova Southeastern University, Fort Lauderdale, Florida, United States of America; 3 Clinical Systems Biology Group, Institute for Neuro-Immune Medicine, Nova Southeastern University, Fort Lauderdale, Florida, United States of America; 4 Department of Psychology and Neuroscience, Nova Southeastern University, Fort Lauderdale, Florida, United States of America; University of Nebraska-Lincoln, UNITED STATES

## Abstract

Sleep is critical for repair as well as the rejuvenation processes in the body and many of these functions are regulated via underlying cellular metabolic homeostasis. Changes in sleep pattern are reported to alter such metabolic function resulting in altered disease susceptibility or behavior. Here, we measured the extent to which overnight total sleep deprivation (SD) in young adult humans can influence systemic (plasma-derived) redox-metabolism including the major antioxidant, glutathione as well as DNA methylation levels. Nineteen participants (n = 19, μ age = 21, SD = 3.09) underwent morning testing before and after overnight total SD. Biochemical measures before and after SD revealed that glutathione, ATP, cysteine, and homocysteine levels were significantly reduced following one night of sleep deprivation (all p’s < 0.01). Parallel to the well-recognized fact that sleep deprivation (maintaining wakefulness) uses up metabolic reserves, we observed that morning cortisol levels were blunted after sleep deprivation. There were no significant correlations between self-reported or actigraphy-measured sleep and the biochemical measurements, strongly indicating that prior sleep behavior did not have any direct influence on the biochemical measures taken at baseline or after sleep deprivation. Results from the current investigation supports the previous literature implicating the induction of oxidative stress and ATP depletion with sleep deprivation. Furthermore, such altered antioxidant status can also induce downstream epigenetic changes. Although we did not measure the specific genes that were altered under the influence of such sleep deprivation, such epigenetic changes could potentially contribute towards disease predisposition.

## Introduction

Sleep is critical for repair as well as the rejuvenation processes in the body such as muscle repair and hormone regulation. A growing body of evidence suggests that sleep plays a critical role in metabolic and energetic recovery. Indeed, sleep loss has been shown to result in adverse metabolic consequences, reduction in anabolic hormones, and increased infection susceptibility[1] (Knutson et al., 2007).

It is possible that sleep deprivation can influence metabolic activities though changes in oxidative processes. For example, a systemic elevation of reactive oxygen species (ROS) levels has been reported in non-human animals after sleep deprivation[2] (Villafuerte et al., 2015). Accordingly, it has been reported that sleep may provide an antioxidant function by eliminating free radicals or ROS produced during wakefulness [3] (Gopalakrishnan et al., 2004). Oxidative stress is defined as an imbalance in the normal equilibrium between formation of reactive oxygen species and antioxidant defense mechanisms. Antioxidants belong to structural heterogeneous groups that share the ability to scavenge free radicals and are the first defense against the potential damage of ROS.

An essential endogenous antioxidant is glutathione (GSH)- a tripeptide (L-[gamma]-glutamil-L-cysteinyl-glycine) found in virtually all cells which, in addition to a variety of other functions, has a central role in protecting cells against damage produced by free radicals [4](Ballatori et al., 2009). Reductions in glutathione levels are commonly used as a marker of oxidative stress [5,6](Trivedi et al., 2014a, Trivedi and Deth, 2012). GSH synthesis occurs through the activity of glutathione synthase and such energy for GSH synthesis is supplied by ATP, and hence, any changes in the energy demand or ATP formation can lead to altered GSH synthesis and antioxidant capacity of the cells, consequently resulting altered redox status in the cells. The synthesis of ATP produces free radicals and reactive oxygen species that are controlled by antioxidant molecules. Wakefulness involves higher metabolism to maintain neuronal electrical potentials, which requires increased oxygen, resulting in a significant production of oxidants and higher usage of ATP [7] (Pena et al., 1999). It is possible, therefore, that ROS and other oxidative stress markers accumulate in the brain tissue during wakefulness and act as somnogenic factors [2,8] Hence, there is a tight interdependence between circadian rhythm, energy production, and redox metabolism.

**Fig 1:**
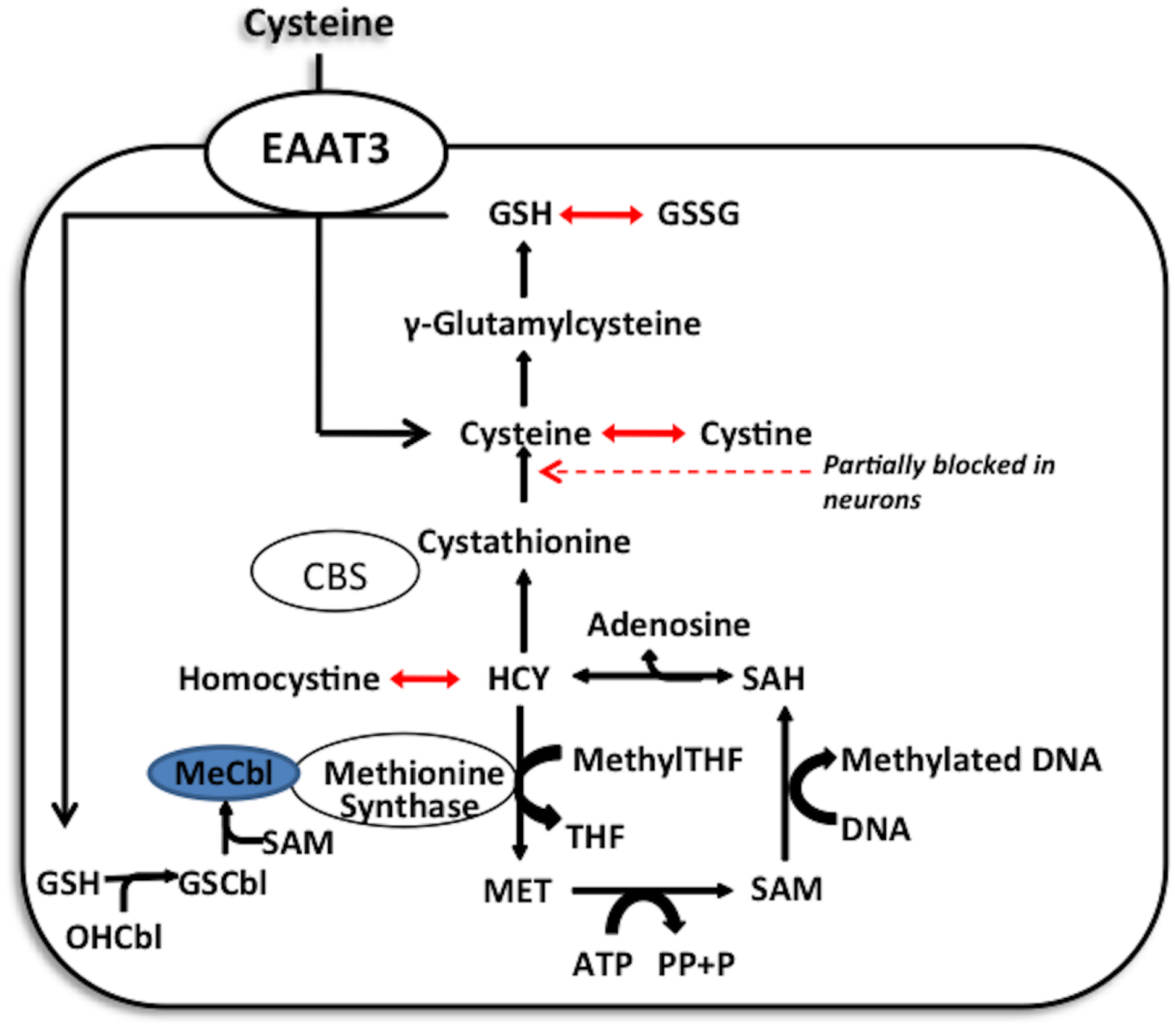
Redox-methylation pathway. Methionine synthase contains a redox-active methylcobalamin cofactor. Under oxidative stress, this cofactor becomes oxidized, limiting methionine synthase activity and can affect the levels of global DNA Methylation Under these conditions, homocysteine can be condensed with serine to form cystathionine and then with cysteine to support GSH synthesis. Another source of the cysteine is through the Excitatory Amino Acid Transport (EAAT3), which is the major source of cysteine especially in the neuronal cells. Cellular redox state is indicated by the GSH/GSSG ratio.

Fig 1 illustrates the two possible pathways that can provide the necessary cysteine for GSH synthesis in any cells: 1) conversion of homocysteine (HCY) to cysteine; 2) transport of extracellular cysteine via EAAT3. Synthesis of cysteine from homocysteine is termed the transsulfuration pathway. About 50% of the cysteine needed as a precursor for the production of GSH in liver is supplied by the transsulfuration pathway [9,10]. Homocysteine can also be utilized to synthesize methionine, via the activity of methionine synthase. The redox status of the cells can control the methionine synthase activity and, in turn, control the methylation reactions including DNA methylation [11,12]. Recent clinical evidence indicates that sleep deprivation can induce elevated levels of DNA methylation of circadian clock genes in tissues with higher metabolic activity (adipose and skeletal muscle) [13,14]. The 5-methylcytosine epigenetic marker can get oxidized to a 5-hydroxymethylcytosine marker under the influence of oxidative stress. Accordingly, we measured both of these changes in the plasma samples on the circulating, free DNA.

In general, although several studies have measured the oxidative stress under sleep deprivation in the central nervous system using animal models, to our knowledge, no previous studies have focused on measuring the effects of sleep deprivation on plasma levels of these antioxidant metabolites as well as the epigenetic status in human studies. Hence, the current study builds on a few reports indicating that sleep loss is specifically associated with an increase in peripheral metabolic rate [14]. Accordingly, we aimed to show (i) the influence of sleep deprivation on systemic metabolism including redox metabolites (ROS, GSH, HCY, and ATP), (ii) and concurrent effects of sleep deprivation on peripheral plasma global DNA methylation levels. We also examined the relationship between sleep deprivation, cortisol levels, and our metabolic biomarkers since (i) sleep deprivation can impact cortisol levels and (ii) such changes in cortisol levels lead to altered redox changes including changes in the GSH levels, as well as epigenetic changes including DNA methylation [15,16]. Finally, in order to ensure that any observed changes were due to the effects of SD and not ongoing sleep loss, we tested the extent to which actigraphy sleep measures related to our biochemical measures. Overall, we provide contributing clinical evidence supporting the interdependence between sleep and redox metabolism.

## Methods

### Participants

The recruitment and testing procedures were approved by and carried out according to a protocol submitted to the Nova Southeastern University (NSU) Institutional Review Board. Upon arrival to the study, participants first read and signed a written informed consent. Nineteen adult participants (n = 19, 10 males, μ age = 21, SD = 3.09) underwent testing after a baseline night and after overnight total sleep deprivation. Following consent, all participants filled out the Epworth Sleepiness Scale (ESS) as a pre-screening tool with an enrollment criterion score of 10 or less. No participants were excluded based on this criterion. In addition, participants were asked to refrain from caffeine intake at least 24 hours before testing. Participants were compensated with a $100 Visa gift card for their time.

### Sleep monitoring and sleep deprivation

Testing occurred between 7:00–9:00 am and included one baseline testing session and one sleep deprivation testing session seven days later. Total sleep time was calculated through the use of Actiwatch wrist monitors and Actiware software (Phillips Respironics, New Jersey). Actiwatch data were also used to verify that participants were awake during the day of the sleep deprivation day before they arrived to the laboratory. During the sleep deprivation evening the participants came to the laboratory at 9:00 p.m. for overnight total sleep deprivation. The participants were constantly monitored by 2–4 researchers throughout the evening. In addition, all participants wore activity monitors throughout the sleep deprivation period. During sleep deprivation the participants were permitted to engage in non-stressful activities (e.g. talking, board games etc.). Only water was permitted during the sleep deprivation period (no other beverages or food was permitted).

### Measures and procedure

At each testing session the participants provided saliva samples for cortisol quantification. Saliva was also collected from each participant by unstimulated passive drool for salivary cortisol analysis (participants drooled directly into a 1.5 mL microcentrifuge tube through a small sterile cylinder). Following this, 3 mLs of blood was taken through venipuncture. Blood was collected into EDTA coated tubes. Immediately after collection, the tubes were centrifuged for 10 minutes at 1,500 x g at 4°C. The plasma was then apportioned into 0.5 ml aliquots and stored at –20°C until analyses were conducted.

### Isolation and measurement of intracellular thiol metabolites

Plasma samples were thawed on ice, and a 10% homogenate was prepared as previously described[17,18]. 100 microL of sample was separated for protein measurements and the remaining lysate was added to a microcentrifuge tube and an equal volume of 0.4 N perchloric acid was added, followed by incubation on ice for 5 minutes. Samples were centrifuged at 13,000g and the supernatant transferred to new microcentrifuge tubes. Using the respective ELISA kits as previously described for Homocysteine, cysteine and glutathione, we performed the measurement of the different thiol metabolites.

### DNA isolation

Genomic DNA was isolated from samples at different time-points for measurement of global DNA methylation as previously described [6,12]. DNA was isolated from the respective plasma samples using the FitAmp Plasma DNA Extraction Kit from Epigentek (Farmingdale, NY). The Maufacturer’s website provides the full protocol. The isolated DNA was cleaned for any contaminating RNA by treatment with RNAase enzyme and quantified using ND-1000 NanoDrop spectrophotometer (Thermo Scientific).

### Global DNA methylation measurements

Global DNA Methylation analysis was performed using the MethylFlash Methylated DNA Quantification Kit from Epigentek (see the manufacturer’s website for full protocol). 100 ng of clean genomic DNA isolated from samples at different time-points, was used for this purpose. DNA-Methylation was quantified using 5-methylcytosine monoclonal antibodies by an enzyme-linked immunosorbent assay–like reaction as previously described [6,12]. The levels of methylated DNA were calculated using the intensity of optical density on a microplate reader at 450 nm. Results were normalized against a standard curve prepare according the manufacturer’s protocol using the given 0–100% methylated standards.

### Determination of ATP

ATP levels were measured with a colorimetric/fluorometric assay kit (BioVision). Samples were processed and analyzed according to the manufacturer’s protocol. Standard curve was generated and sample absorbance was measured accordingly.

### Cortisol quantification

Our cortisol quantification methods were carried out as previously described [19]. The tubes were placed in a freezer following participant testing and stored at −20°C. Cortisol was quantified through the use of a commercially available human enzyme immunoassay (EIA) kit (Salimetrics LLC, USA) which has a 0.91correlation with serum and a sensitivity < 0.007 ug/dL. Interpolated concentrations are in ug/dL.

### Follow up correlation analyses

We found that the baseline average sleep duration was lower than expected (see Results). This prompted us to order carry out a follow-up analysis on self-report and actigraphy sleep measures in order determine if the low average sleep duration (prior to SD) had any bearing on our biochemical measures. To that end, we conducted a correlation analysis to estimate the relation between self-report sleep measures and lab results, while regression analysis was used to predict the effect of objective sleep quality on the biochemical measures. Spearman rank correlation was assessed between individual biochemical measures and self-report measures on the Pittsburgh Sleep Quality Index and the Insomnia Severity Index. Correlation analysis was performed using the corr function in MATLAB (R2014b, MathWorks, Inc.). Due to the number of correlations calculated the false discover rate (FDR) was calculated for each correlation from the resulting p-values using the procedure introduced by Storey (Storey, 2002) using the *mafdr* function in MATLAB (R2014b, MathWorks, Inc.). Correlation values with FDR of less than 0.05 were taken to be reliably significant. To assess the effect of objectively measured sleep quality on lab results the responses of each of the biochemical measures were independently fit to a linearized model of Actiwatch measures of Total Sleep Time (TST), Sleep Onset Latency (SOL), and Sleep Efficiency (SE). The models were generated using the *fitlm* function in MATLAB (R2014b, MathWorks, Inc.). Models were considered significant for p < 0.05, and taken to indicate a predictive relation between sleep quality and biochemical measures at baseline or after sleep deprivation.

### Statistical analyses

The effect of sleep deprivation on each of our biochemical measures were individually analyzed by paired samples t-tests comparing results before and after sleep deprivation (between testing sessions). Since we tested both men and women in our study we also carried out between samples t tests in order to check for possible sex differences in response to sleep deprivation. All calculations were conducted using an SPSS statistical package (version 19, SPSS inc., IBM). All reported p values are two-tailed with an a priori significance level of p < 0.05. All the raw data is provided in the supplemental information as a supplemental S1 Table.

## Results

### Actigraphy

**Fig 2:**
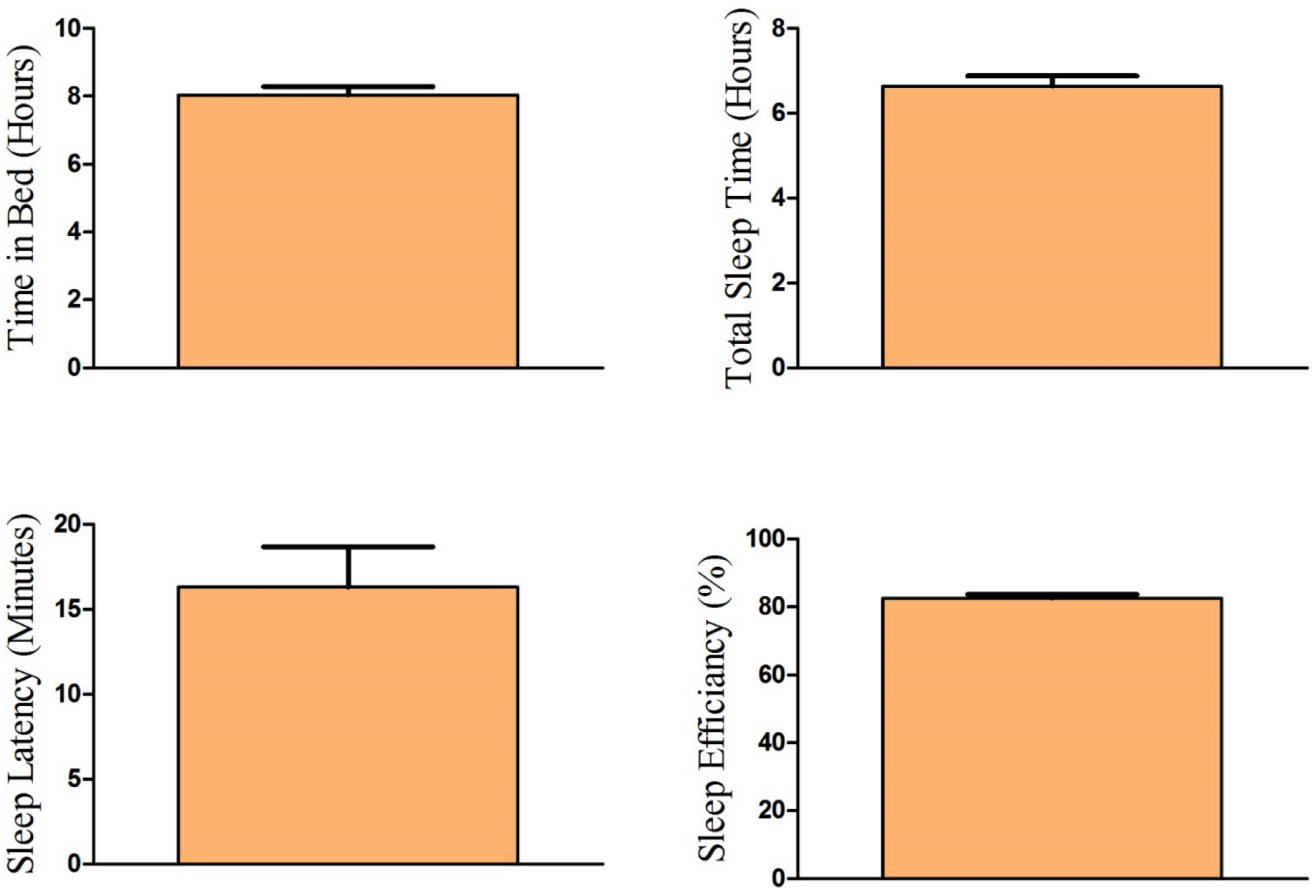
Actigraphy measures. Sleep behavior was objectively verified the week prior to sleep deprivation through actigraph monitoring. Top left: actigraphy-recorded time-in-bed confirmed that the participants generally adhered to the instruction to sleep 8 hours the week prior to sleep deprivation (mean = 8.03, SD = 1.12). Top Right: Total sleep actigraphy-recorded sleep time was 6 hours and 10 min (SD = 1.04). Bottom Left: The average sleep latency was 16 minutes (SD = 10.29). Bottom Right: the average sleep efficiency was 82.64% (SD = 4.71).

Actigraphy data are shown in Fig 2. Although participants were instructed to sleep 8 hours each night prior to sleep deprivation, sleep behavior was still objectively verified the week prior to sleep deprivation through actigraphy monitoring in order to ensure that the participants were not experiencing sleep loss the week prior to experimental sleep deprivation. The average actigraphy-recorded time in bed confirmed that the participants adhered to this instruction (mean = 8.03, SD = 1.12). However, despite having 8 hours of time in bed, actigraphy recording also showed that the total sleep time was only 6 hours and 10 min (SD = 1.04). The average sleep latency was 16 minutes (SD = 10.29) and the average sleep efficiency was 82.64% (SD = 4.71). Although the total time in bed was in line with typical habitual sleep patterns [20–22]. There was no effect of sex on any of the actigraphy measures (all p’s > 0.05).

### Biochemical measures

**Fig 3:**
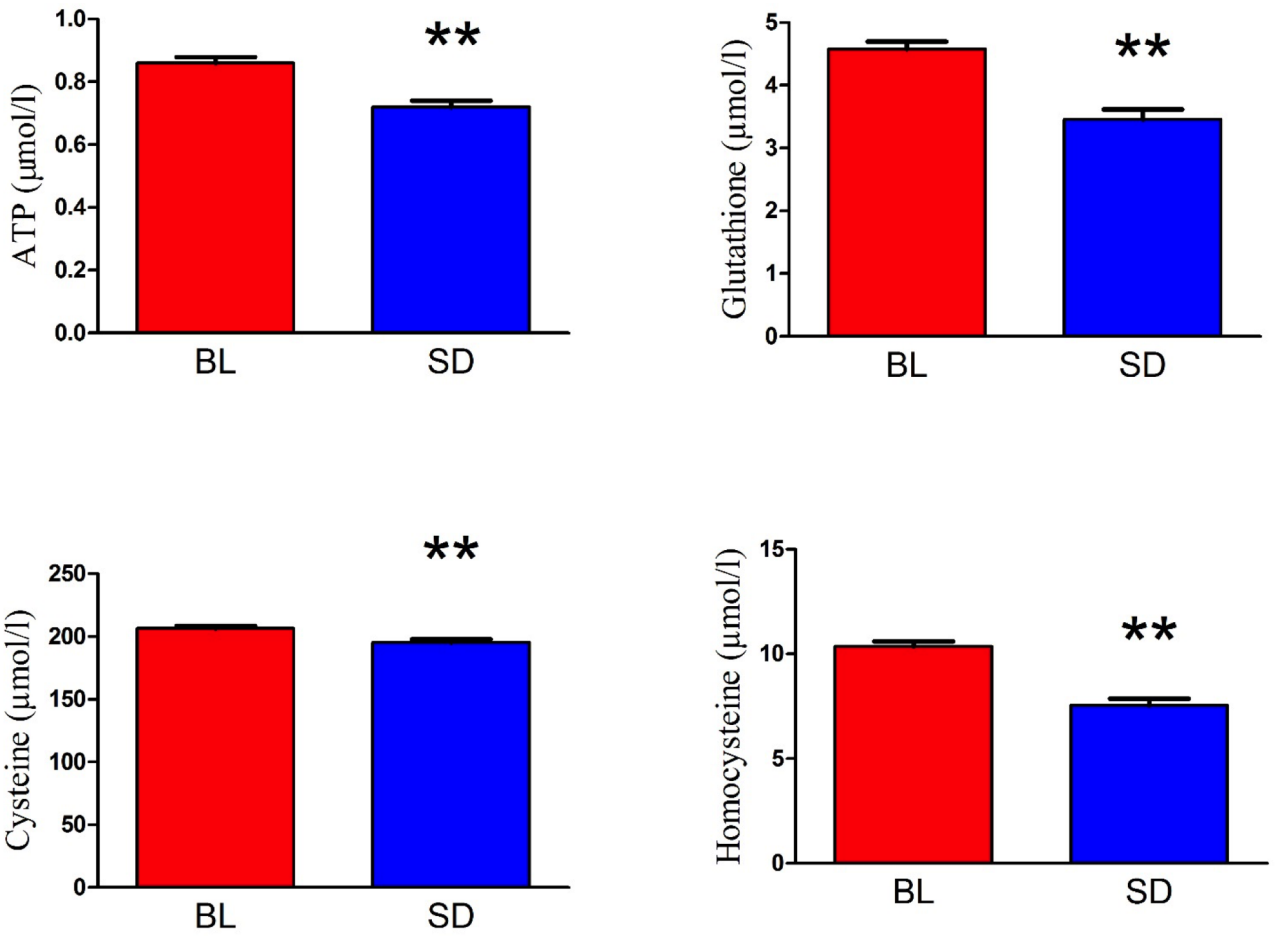
Thiol metabolite levels in plasma. Thiol / Thioether metabolite levels were analyzed via HPLC with electrochemical detection before (BL) and after sleep deprivation (SD) in participants (Mean +/- SEM). GSH / GSSG ratio indicates oxidative stress. HCY—homocysteine, Cys- cysteine were measured in uM. ATP measurement. ATP was measured using commercially available kit and expressed in umol/ L of plasma. Asterisk indicates a significant difference from baseline * = p < 0.05, ** = p < 0.01.

**Fig 4:**
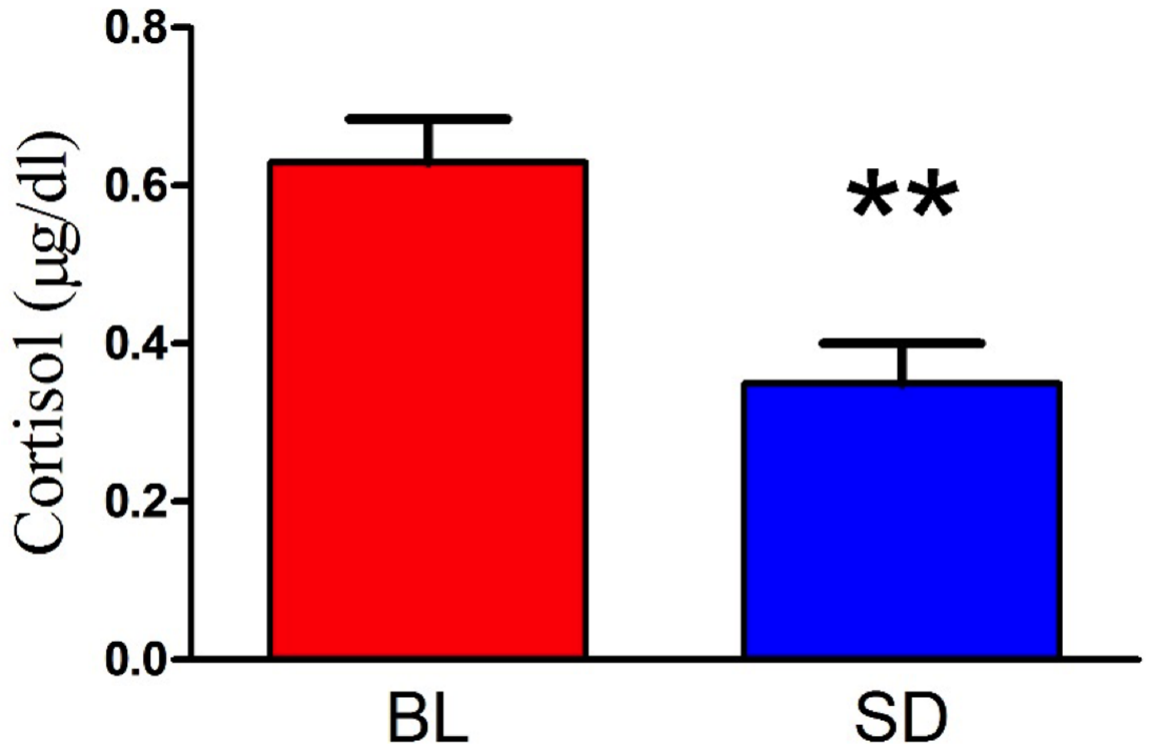
Cortisol. Moring cortisol levels were significantly lower after sleep deprivation (mean = 0.35, SD = 0.22) relative to the morning of baseline testing (mean = 0.63, SD = 0.24), t(18) = 6.61, p < 0.01.

Plasma-derived biochemical measures before and after sleep deprivation can be seen in Fig 2. Paired samples t tests revealed that relative to baseline (mean = 0.86, SD = 0.07), ATP levels were significantly reduced following one night of sleep deprivation (mean = 0.72, SD = 0.09), t(18) = 6.67, p < 0.01. After sleep deprivation, cysteine (mean = 195.24, SD = 11.53) levels were also significantly reduced relative to baseline (mean = 206.24, SD = 8.67) t(18) = 3.69, p < 0.01. Similarly, sleep deprivation also resulted in reduced homocysteine (mean = 7.55, SD = 1.38) levels that were reduced relative to baseline (mean = 10.35, SD = 1.38) t(18) = 6.25, p < 0.01. In parallel to these results, GSH levels were also lower after sleep deprivation (mean = 3.46, SD = 0.69) relative to baseline (mean = 4.58, SD = 0.51) t(18) = 8.40, p < 0.01; see Fig 3. Lastly, relative to the morning of baseline testing (mean = 0.63, SD = 0.24), cortisol levels were significantly lower following sleep deprivation (mean = 0.35, SD = 0.22), t(18) = 6.61, p < 0.01 (see Fig 4). There was no effect of sex on the biochemical measures, except for a sex difference in GSH at baseline only. Men (mean = 4.81, SD = 0.36) had higher levels than women (mean = 4.33, SD = 0.54), t(17) = 2.27, p < 0.05. We followed up on this by independently analyzing men and women on GSH. When analyzed separately, both the male and the female groups still show a significant reduction in GSH after sleep deprivation: males t(9) = 8.00, p < 0.01 females t(8) = 4.62, p < 0.01.

### DNA methylation

**Fig 5:**
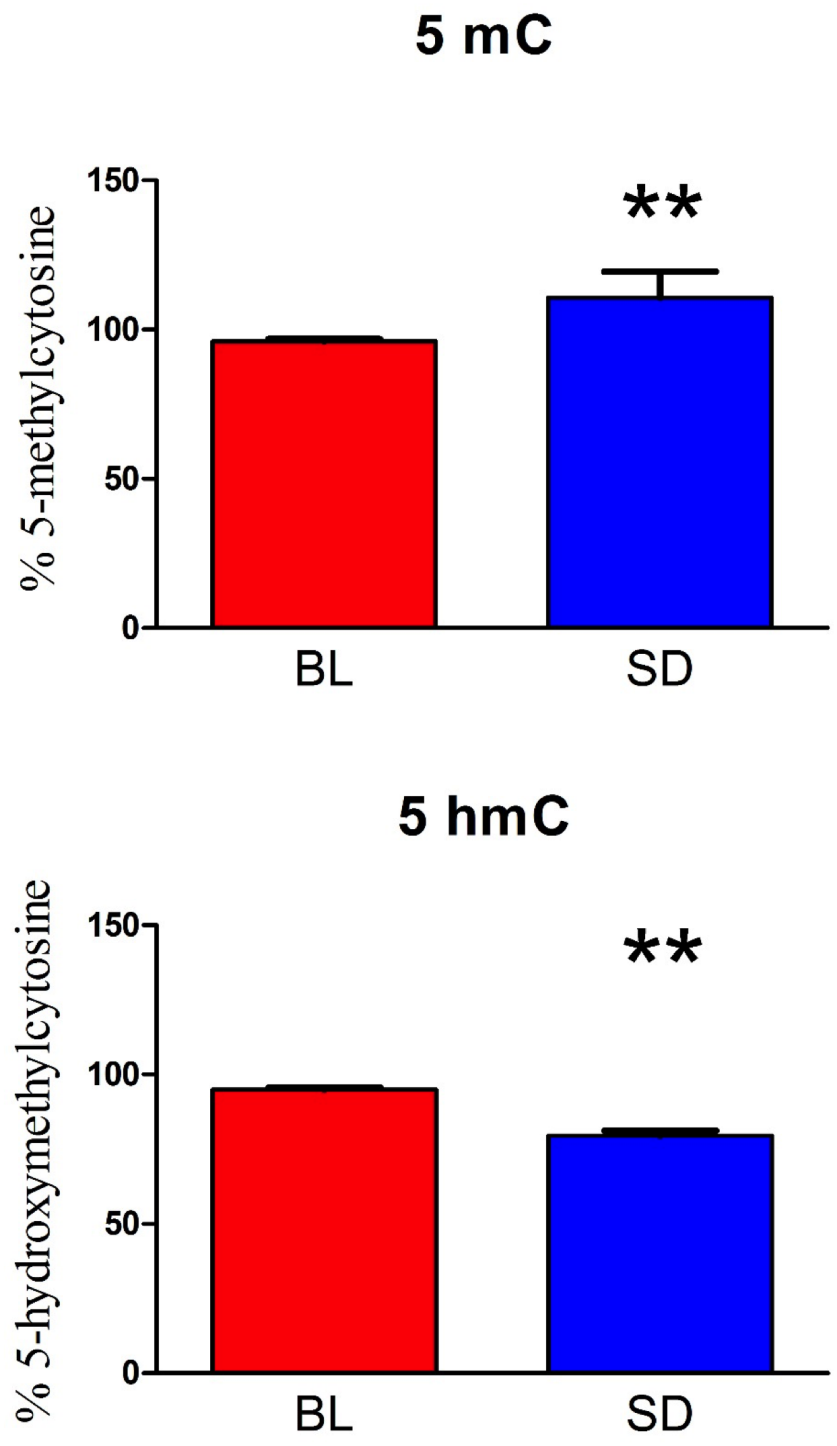
DNA methylation levels. Plasma samples were used to isolate cell free circulating DNA. The percentage of 5-methylcytosine and 5-hydroxymethylcytosine on isolated DNA was measured using an ELISA assay (Mean +/- SEM). Asterisk indicates a significant difference from baseline * = p < 0.05, ** = p < 0.01.

As shown in Fig 5, our global DNA methylation studies indicated altered levels of both 5-methylcytosine t(18) = -10.63, p < 0.01 as well as 5-hydroxymethylcytosine t(18) = 6.25, p < 0.01 following sleep deprivation. In particular, sleep deprivation resulted in elevated 5-methylcytosine (mean = 110.74, SD = 8.71), relative to baseline (mean = 96.05, SD = 3.08) and decreased levels of 5 hydroxymehtylcytosine (mean = 79.58, SD = 7.17), relative to baseline levels (mean = 94.95, SD = 3.17). There was no effect of sex on 5-methylcytosine or 5-hydroxymethylcytosine (all p’s > 0.05).

### Associations between sleep and biochemical measures

Spearman rank correlation analysis revealed no significant correlation between self-reported or actigraphy-measured sleep and the biochemical measurements. The generation of linear models of sleep quality measures describing independent biochemical measures also resulted in no significant relations. This indicates that prior sleep behavior, whether normal (TST 7–9 hours, SOL 15–20 min, SE > 85%) or dysfunctional, did not have any bearing on the biochemical measures taken at baseline or after sleep deprivation. Spearman rank correlation analyses aimed at relating cortisol levels to our metabolic variables before and after sleep deprivation showed a significant correlation between cortisol levels at baseline and ATP at baseline (r_s_ (19) = -0.55, p < 0.05). This relationship was no longer apparent after sleep deprivation (r_s_ (19) = -0.19, p > 0.05).

## Discussion

The current study showed that acute sleep deprivation results in redox-based global DNA methylation changes in human plasma samples. Specifically, we found that the levels of plasma antioxidant GSH are decreased with a concomitant elevation in oxidative damage. Additionally, we also observed depleted levels of ATP which can indicate altered mitochondrial functioning. Such altered redox and epigenetic status, mitochondrial function, and depleted ATP levels are of great importance, especially since previous studies show such changes to be a significant contributor to neurological diseases including neurodegenerative diseases [23,24]. Acute sleep deprivation also altered the levels of cysteine and homocysteine and such altered levels are known to be a risk factors for several neurological disorders [25,26]. Thus, these results support previous reports emphasizing neurological manifestations of sleep loss [23,24,27,28]. Hence, the antioxidants (and the precursors to antioxidant synthesis via the transulfuration pathway) are altered under the influence of sleep deprivation. This is not surprising, given that sleep is known to be a replenisher for redox metabolites and changes in sleep pattern in form of sleep deprivation might restrict this ability.

We have previously shown that decreased GSH levels and elevated oxidative stress can subsequently result in altered epigenetic changes, specifically DNA methylation levels via the vitamin-B12-dependent enzyme methionine synthase [29]. In agreement, we observed that sleep deprivation increased the global DNA methylation levels. Such altered redox-status and DNA methylation status is prevalent in several different neurological disorders, including in the pathology of neurodegenerative diseases [30]. Hence, combined with the results on the antioxidant status, our results validate previous preclinical findings indicating that sleep loss is a form of systemic cell injury that can result in uncompensated oxidative stress [31,32]. Incomplete antioxidant protection, as measured in the current study, could result in injured cells that then would either be repaired with adaptive molecular epigenetic changes, or if uncontrolled, could result in apoptosis and cell death. It is important to note that the GSH measures were higher in men than women at baseline. However, sleep deprivation-induced reductions in GSH levels eclipse sex differences at baseline; therefore, the effect of sleep deprivation on GSH is not driven by sex differences. This is also supported by the finding that, when analyzed as separate groups, both groups show a significant reduction in GSH after baseline

Importantly, we found that the metabolic biomarkers were not associated with self-reported or actigraphy measured sleep patterns despite the average sleep time of 6 hours and 10 minutes the week prior to sleep deprivation. This indicates that prior sleep behavior did not have any bearing on the biochemical measures after sleep deprivation. In other words, it was the isolated consequences of overnight total sleep deprivation that influenced the above metabolic and molecular changes, not a combination of accumulated prior sleep behavior and sleep deprivation. Although we did not characterize any changes for recovery after the sleep deprivation phase, the current study suggests that even a single episode of sleep deprivation has profound impact on redox metabolites. The potential recovery after such sleep deprivation in the form of underlying adaptive molecular changes (in the form of specific genes / epigenetic status of genes) still remains to be characterized

Of note, however, we did find that baseline cortisol levels were associated with ATP levels at baseline, but not after sleep deprivation. At baseline, lower cortisol levels were associated with higher ATP levels. This is consistent with previous reports indicating that cortisol stimulates gluconeogenesis which consumes ATP [33]. Following sleep deprivation, however, the relationship between cortisol levels and ATP levels was no longer apparent. Cortisol levels were measured in the morning when cortisol levels are normally highest (known as the cortisol awakening response, CAR), in order to meet the energy demands of waking and starting the day [34,35]. After one night of sleep deprivation, morning cortisol levels were significantly lower relative to baseline levels. This finding parallels previous research that found a decrease in cortisol after sleep deprivation [36,37]. It is possible that increased negative feedback regulation of the hypothalamic-pituitary-adrenal (HPA) axis as a consequence of increased cortisol release associated with maintaining wakefulness[38] results in a shunting of the morning CAR. Increased negative feedback regulation of the HPA axis would also explain the finding that the correlation between cortisol and ATP use is no longer apparent after sleep deprivation.

The current study provides an initial, unique perspective of imbalance in redox-methylation pathway and underlying molecular changes in the form of epigenetic status, which need to be validated in a larger cohort. Although we did not investigate any transcription changes, the current study serves as a platform to warrant further investigation into effects of sleep deprivation on specific gene expression and how the body compensates for the sleep loss with underlying adaptive metabolic and molecular changes in form of redox and epigenetic status respectively. Future studies characterizing the transcriptome and genome-wide epigenetic studies could be conducted to define the phenotypic and molecular consequences occurring due to acute sleep deprivation.

## Conclusion

The results from the current investigation clearly implicate the induction of oxidative stress and ATP depletion under the influence of sleep deprivation. Furthermore, the altered antioxidant status was also associated with downstream epigenetic changes in the form of global DNA hypomethylation. Although we did not measure site-specific changes in epigenetic status and gene expression levels, such imbalance in the redox status, antioxidant levels, and global hypomethylation are an underlying contributing symphony for neurological disorders. The significance of these findings is underscored by the fact that redox metabolic replenishers are already in use for treatment of sleep loss. Hence, the current study warrants future investigation for contribution of chronic sleep loss towards elevated risks for neurological disorders with a potential underlying mechanistic contribution of altered redox homeostasis. The current work points to therapeutic targeting modalities for preventing any neurological damage occurring due to sleep deprivation.

## Supporting information

S1 Table Contains all the raw datasets. (XLSX)
